# Supplementary material for: Encapsulation of Vitamin C by Glycerol-Derived Dendrimers, Their Interaction with Biomimetic Models of Stratum corneum and Their Cytotoxicity
Source: Molecules. 2022 Nov 18;27(22):8022. doi: 10.3390/molecules27228022 (PMC9698622; doi:10.3390/molecules27228022)
Supplement: Supplementary file 1 [file molecules-27-08022-s001.zip › molecules-2007191-supplementary.pdf]

Supplementary Materials

Encapsulation of Vitamin C by Glycerol-Derived Dendrimers, Their Interaction with Biomimetic Models of *Stratum corneum* and Their Cytotoxicity

Katia Bacha [1,2], Catherine Chemotti [2], Jean-Claude Monboisse [3], Anthony Robert [1], Aurélien L. Furlan [2], Willy Smeralda [2], Christian Damblon [4], Julien Estager [5], Sylvie Brassart-Pasco [3], Jean-Pierre Mbakidi [1], Jelena Pršić [2], Sandrine Bouquillon [1,*] and Magali Deleu [2,*]

1 Molecular Chemistry Reims Institute, UMR CNRS 7312, Reims Champagne-Ardenne University, Boîte n° 44, B.P. 1039, F-51687 Reims, France
2 Laboratory of Molecular Biophysics at Interfaces (LBMI), Gembloux Agro-Bio Tech-University of Liege, Passage des Déportés, 2 B-5030 Gembloux, Belgium
3 Laboratoire de Biochimie Médicale et de Biologie Moléculaire, UMR CNRS/URCA 7369 (MEDyC), UFR Médecine, Reims Champagne Ardenne University, 51 Rue Cognacq Jay, F-51095 Reims, France
4 Structural Biological Chemistry Laboratory, MolSys Research Unity, University of Liege, 11, Allée du six Août, 4000 Liège, Belgium
5 Certech, Rue Jules Bordet, 45-Zone Industrielle C, B 7180 Seneffe, Belgium
* Correspondence: sandrine.bouquillon@univ-reims.fr (S.B.); magali.deleu@uliege.be (M.D.)

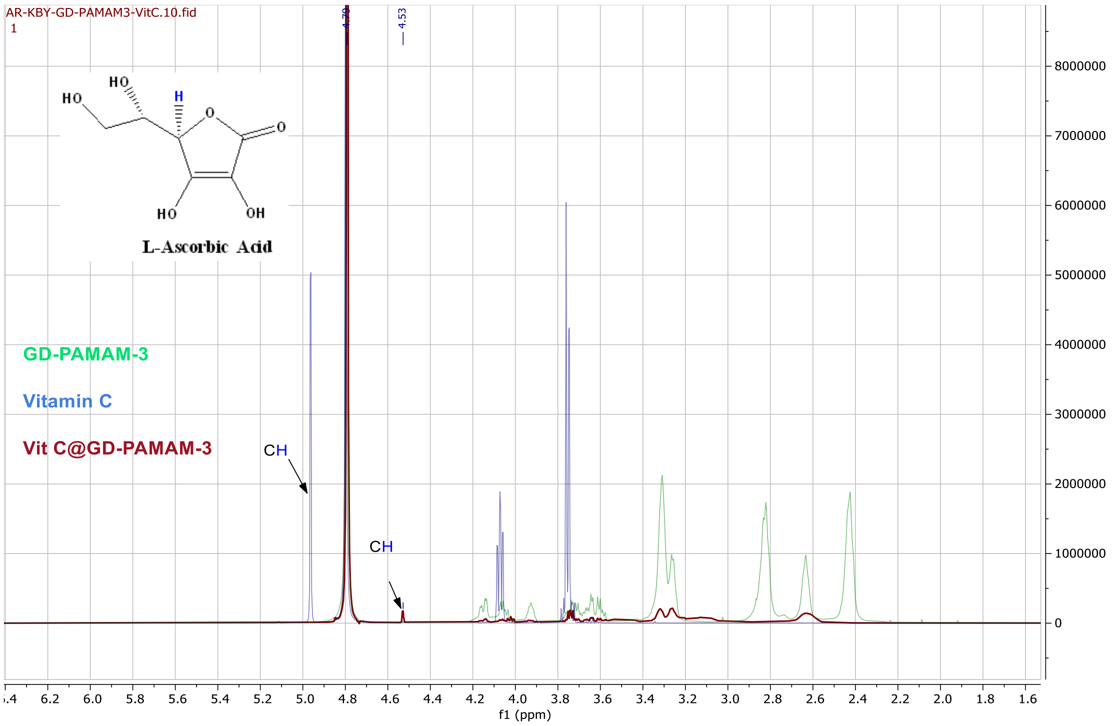

Figure S1. ^{1}H NMR of GD-PAMAM-3 (green), Vitamin C (blue) and Vit C@GD-PAMAM-3 (red) in D_2O.

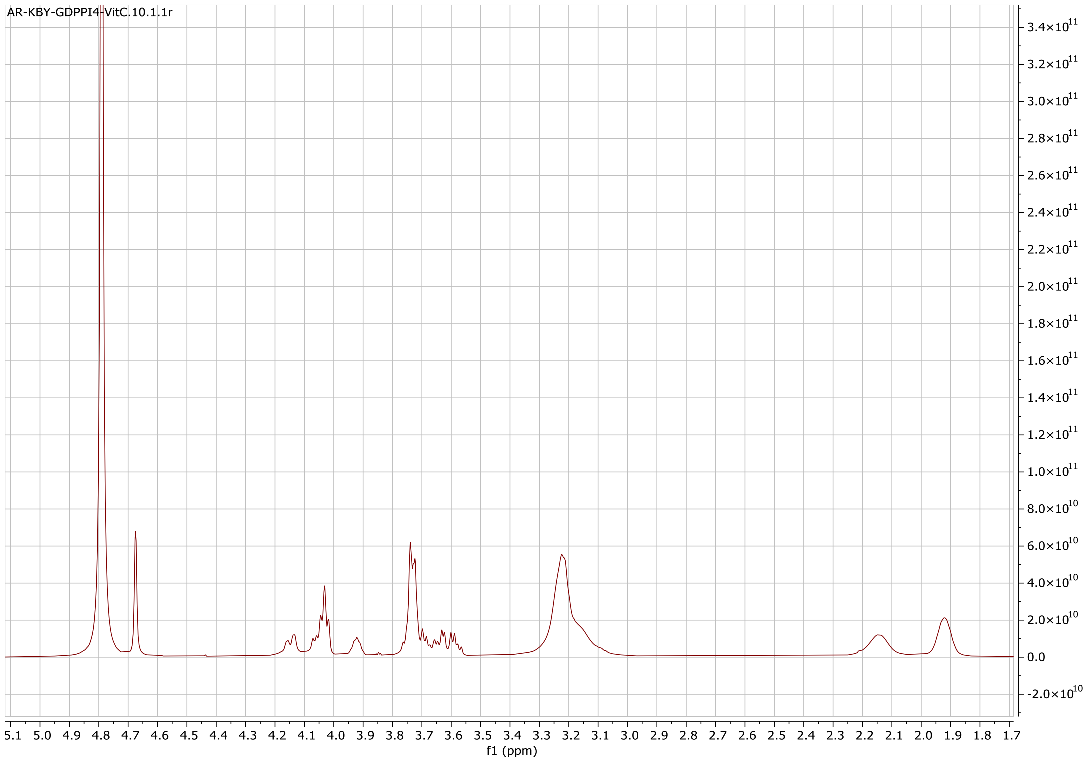

Figure S2. ^{1}H NMR of Vit C@GD-PPI-4 in D_2O.

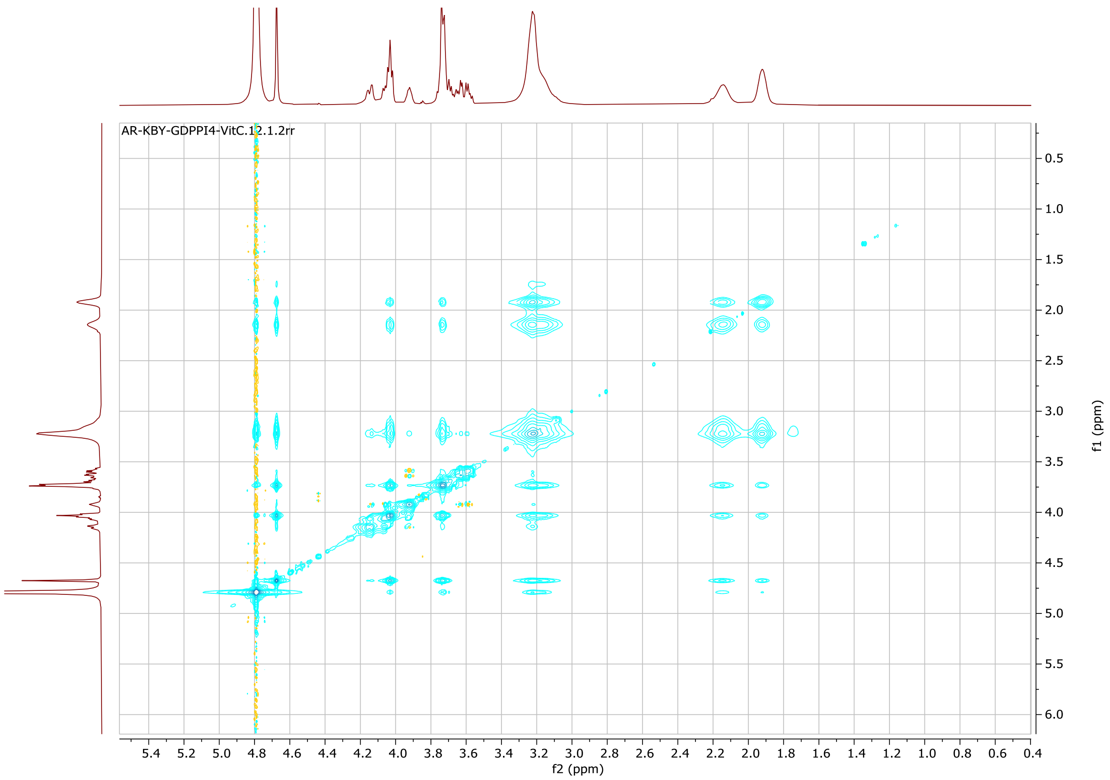

Figure S3. Vit C@GD-PPI-4 NOESY NMR shows NOE interactions between the protons of vitamin C (3.73, 4.03 and 4.64 ppm) with those of the GD-PPI-4 (1.92, 2.14 and 3.22 ppm) in D_2O.

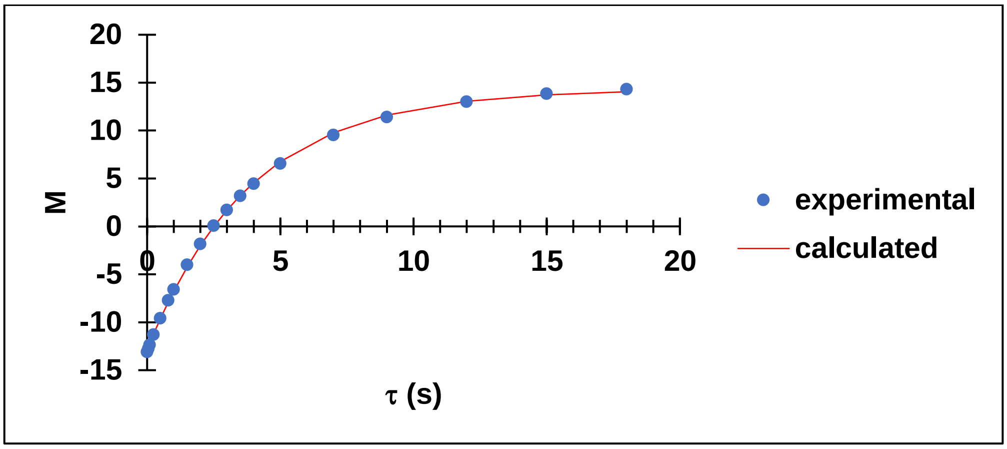

Figure S4. T1 measurements of free Vitamin C: Experimental (blue dot) and calculated (red line) determination of T_1 for the ^{1}H (at 4.95 ppm which have the biggest value of T_1) of free Vitamin C by inversion-recovery pulse sequence. We fit the curve by $\boldsymbol{M} = \boldsymbol{M_0}\left(\mathbf{1} - \boldsymbol{k}\,\boldsymbol{exp}\left(\frac{-\boldsymbol{\tau}}{\boldsymbol{T_1}}\right)\right)$ where $\boldsymbol{M}$ is the magnetization, $\boldsymbol{M_0}$ is the magnetization at $\boldsymbol{\tau} = \mathbf{0}$s, $\boldsymbol{\tau}$ is the delay between the 180° and 90° pulses, and $\boldsymbol{k}$ is the efficiency of 180° pulse (if we have 180° pulse, we must have $\boldsymbol{k} = \mathbf{2}$). We find $\boldsymbol{M_0} = \mathbf{14.3}$, $\boldsymbol{k} = \mathbf{1.9}$ and $\boldsymbol{T_1} = \mathbf{3.9}\ \boldsymbol{s}$ by this NMR sequence with 30 s of recycle delay (delay > 5 × T_1).

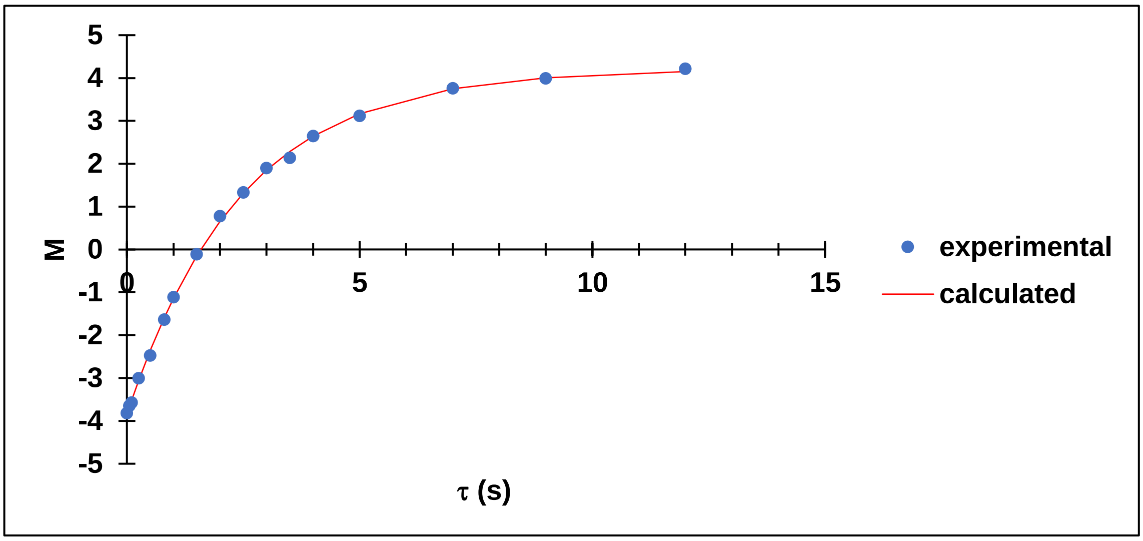

Figure S5. T1 measurements of Vitamin C with GD-PAMAM3: Experimental (blue dot) and calculated (red line) determination of T_1 for the ^{1}H (at 4.53 ppm which have the biggest value of T_1) of Vitamin-C with GD-PAMAM3 by inversion-recovery pulse sequence. We find $\boldsymbol{M_0} = \mathbf{4.2}$, $\boldsymbol{k} = \mathbf{1.91}$and $\boldsymbol{T_1} = \mathbf{2.45}$ s by this NMR sequence with 40 s of recycle delay (delay > 5 × T_1).

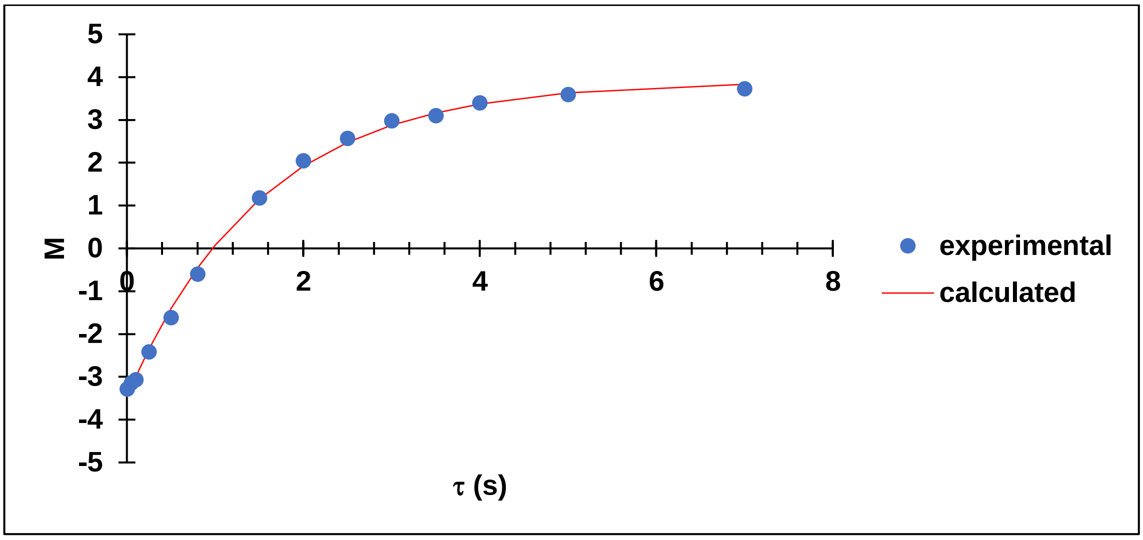

Figure S6. T1 measurements of Vitamin C with GD-PPI4: Experimental (blue dot) and calculated (red line) determination of T_1 for the ^{1}H (at 4.68 ppm which have the biggest value of T_1) of Vitamin C with GD-PPI4 by inversion-recovery pulse sequence. We find $\boldsymbol{M_0 = 3.9}$, $\boldsymbol{k = 1.89}$ and $\boldsymbol{T_1 = 1.5}$ s by this NMR sequence with 40 s of recycle delay (delay > 5 × T_1).

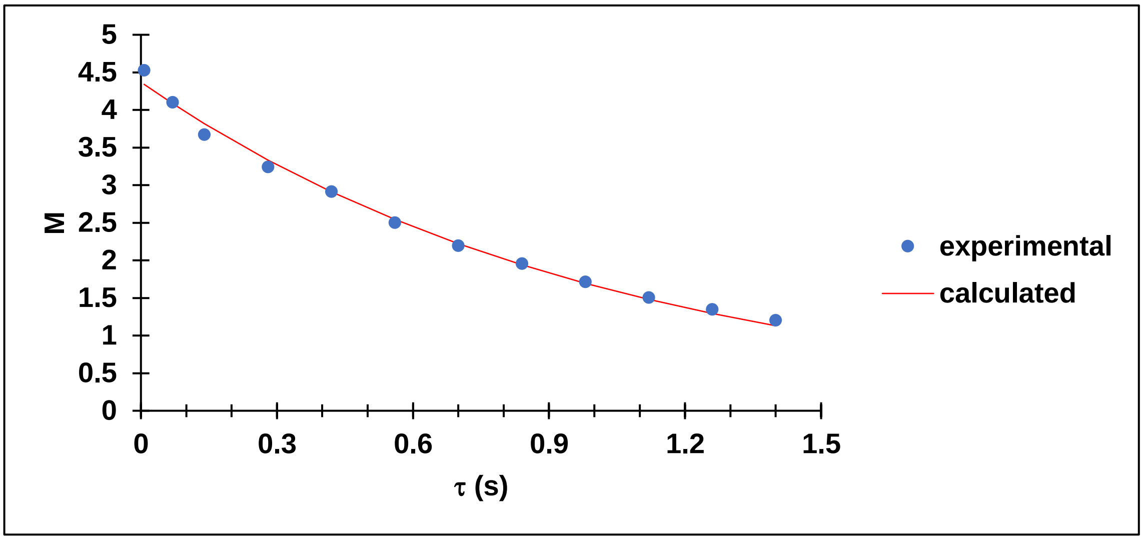

Figure S7. T2 measurements of free Vitamin C: Experimental (blue dot) and calculated (red line) determination of T_1 for the ^{1}H (at 4.08 ppm) of free Vitamin C by CPMG pulse sequence. We fit the curve by $\boldsymbol{M = M_0 exp\left(\frac{-\tau}{T_2}\right)}$ where $\boldsymbol{M}$ is the magnetization, $\boldsymbol{M_0}$ is the magnetization at $\boldsymbol{\tau = 0}$s, and $\boldsymbol{\tau}$ is the delay between the 90° pulse and the beginning of signal acquisition. We find $\boldsymbol{M_0 = 4.37}$ and $\boldsymbol{T_2 = 1}.0$ s by this NMR sequence with 40 s of recycle delay

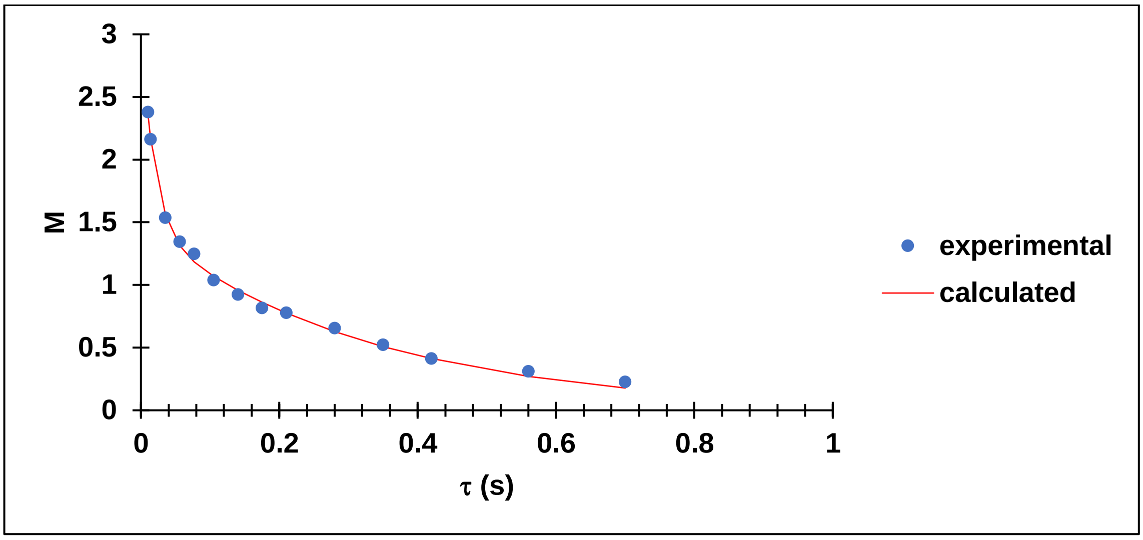

Figure S8. T2 measurements of Vitamin C with GD-PAMAM3: Experimental (blue dot) and calculated (red line) determination of T_2 for the ^1H (at 4.05 ppm) of Vitamin C with GD-PAMAM3 by CPMG pulse sequence. There are two kind of signal (dendrimer and Vitamin-C) so we fit the curve by $\boldsymbol{M = k_d \times M_{0d} \times exp\left(\frac{-\tau}{T_{2d}}\right) + k_v \times M_{0v} \times exp\left(\frac{-\tau}{T_{2v}}\right)}$ where subscripts $\boldsymbol{d}$ and $\boldsymbol{v}$ designate dendrimer and vitamin-C respectively. Finally, we find $\boldsymbol{k_v \times M_{0v} = 1.45}$, and $\boldsymbol{T_2 = 0.33\ s}$ by this NMR sequence with 40 s of recycle delay**.**

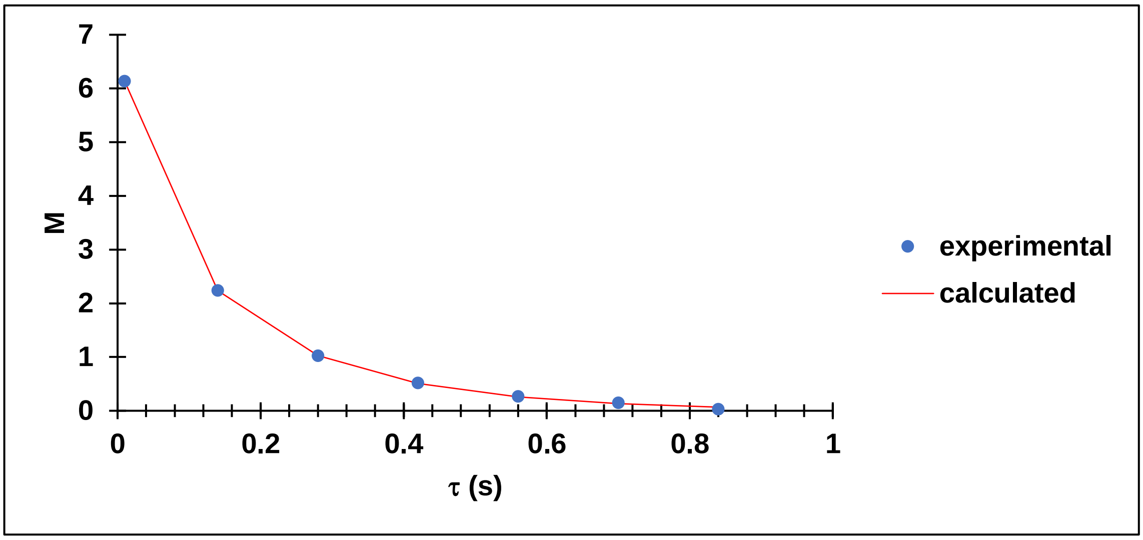

Figure S9. T2 measurements of Vitamin C with GD-PPI4: Experimental (blue dot) and calculated (red line) determination of T_2 for the ^1H (at 4.15 ppm) of Vitamin-C with GD-PPI4 by CPMG pulse sequence. There are two kinds of signal (dendrimer and Vitamin-C). Finally, we find $\boldsymbol{k_v \times M_{0v} = 3.87}$, and $\boldsymbol{T_2 = 0.20}$ s by this NMR sequence with 10 s of recycle delay.

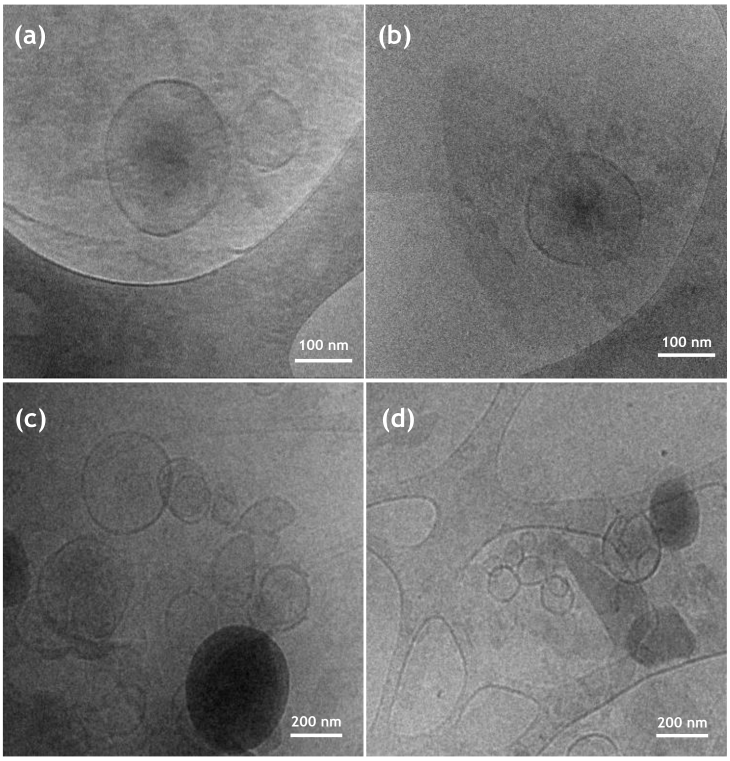

Figure S10. Cryo-TEM images of liposomes at 1.2 mM: (**a**) and (**b**) in absence of GD-PAMAM-3 showing few and isolated Large unilamellar vesicles (LUVs), (**c**) and (**d**) in presence of GD-PA-MAM-3 at 0.48 μM showing some liposome aggregation.
